# A SYSTEMATIC METANALYSIS ON DIAGNOSTIC VALUE OF DsG3 ELISA FOR PEMPHIGUS VULGARIS

**DOI:** 10.4103/0019-5154.53181

**Published:** 2009

**Authors:** Viroj Wiwanitkit

**Affiliations:** *From Wiwanitkit House, Bangkhae, Bangkok, Thailand 10160. E-mail: wviroj@yahoo.com*

Sir,

Pemphigus vulgaris is an important dermatological immune disorder. It presents with blistering skin lesions. Recently, enzyme-linked immunosorbent assay (ELISA) using desmoglein (Dsg) 3 has become the new diagnostic tool for this skin disease. The diagnostic value of Dsg3 ELISA test is varied in different settings. In addition, most reports on the diagnostic value of Dsg3 ELISA contained only a few subjects. Therefore, a systematic metanalysis on this topic is required. In this paper, the author summarizes the diagnostic value of Dsg3 ELISA for pemphigus vulgaris. There are four quoted papers[1–4] on this topic. There are 232 cases and 446 controls [Table 1]. According to this study, the overall sensitivity and specificity are equal to 90.5 and 98.7%, respectively. This can imply the good diagnostic property of Dsg3 ELISA for pemphigus vulgaris.

**Table 1:** Reports on diagnostic value of Dsg3 ELISA for pemphigus vulgaris

Authors	Number of subject		Sensitivity (%)	Specificity (%)
			
	Cases	Controls		
Huang *et al*.[1]	20	114	85	99.1
Harmann *et al*.[2]	82	77	95	98
Amagai *et al*.[3]	81	179	85.2	100
Ishi *et al*.[4]	49	76	94	96
